# Comment on Piscitelli et al. Hospitalizations in Pediatric and Adult Patients for All Cancer Type in Italy: The EPIKIT Study under the E.U. COHEIRS Project on Environment and Health. *Int. J. Environ. Res. Public Health* 2017, *14*, 495

**DOI:** 10.3390/ijerph14080919

**Published:** 2017-08-16

**Authors:** Benedetto Terracini, Daniela Alessi, Elena Isaevska, Vanda Macerata, Corrado Magnani, Milena Manasievska, Milena Maule, Franco Merletti, Maria Luisa Mosso, Tiziana Rosso, Carlotta Sacerdote

**Affiliations:** 1 Childhood Cancer Registry of Piedmont, Cancer Epidemiology Unit, Department of Medical Sciences, University of Turin and AOU Città della Salute e della Scienza, 10126 Turin, Italy; benedetto.terracini@fastwebnet.it (B.T.); daniela.alessi@cpo.it (D.A.); isaevska.elena@gmail.com (E.I.); vanda.macerata@cpo.it (V.M.); manasievskamilena@gmail.com (M.M.); franco.merletti@unito.it (F.M.); marialuisamosso@libero.it (M.L.M.); tiziana.rosso@cpo.it (T.R.); carlotta.sacerdote@cpo.it (C.S.); 2 Department of Translational Medicine, Unit of Medical Statistics and Cancer Epidemiology, CPO Piemonte and University of Piemonte Orientale, 28100 Novara, Italy; magnani@med.unipmn.it

In the “Epikit study” [1], P. Piscitelli with 27 co-authors from 20 scientific institutions estimated the absolute number of hospital admissions for newly diagnosed cancer in people aged 0–19 in Italian provinces and regions in 2007–2011. On this basis, they calculated standardized hospitalization rates (SHRs) as a proxy for incidence rates.

The authors correctly advocate the use of data produced by traditional population-based cancer registries as a gold standard for “providing official epidemiological estimates”. Nevertheless, they fail to compare their estimates with such a gold standard—an exercise which would have shed some light on the reliability of their estimates. The exercise would have been feasible for Italian populations living in provinces or regions served by a conventional cancer registry, such as Piemonte and the provinces of Parma, Varese, and Ragusa. For these (and other) populations, both incidence rates and the absolute number of cases (numerator of rates) in the six-year period 2003–2008 have been published by the Italian Association of Cancer Registries [2] and are known to the authors (their reference 28).

As a matter of fact, a juxtaposition of the two series of data (cancer registries and hospital admissions for newly diagnosed cancer) suggests that Piscitelli et al. [1] overestimated the number of incident cases. For instance, the article provides an average yearly figure of 245 hospital admissions for newly diagnosed cancer cases in residents in Piemonte aged 0–19, whereas in our Cancer Registry the number of incident malignant tumors per year was 132 in 2003–2008 ([2], their reference 28) and 117 in the quinquennium from 2007 and 2011 (unpublished data). Similarly, in the provinces of Parma, Varese, and Ragusa, Piscitelli et al. report 33, 50, and 24 hospitalizations for newly diagnosed cancers in age 0–19 per year [1], whereas figures published by cancer registries were respectively 13.8, 26.3, and 8.8 [2]. Considering these differences, it is surprising that in the discussion, the authors focus only on the possibility of an underestimation (caused by cancer patients who are not hospitalized cases) and do not mention any source of overestimation.

Curiously enough, and in spite of the overestimation of incident cases, SHR rates are remarkably low. Table 2 of the paper [1] provides annual standardized SHR rates of 5.90, 8.23, and 8.85 per 100,000 in Northern, Central, and Southern Italy, respectively. It is difficult to reconcile these figures with the corresponding rate estimated on the pool of Italian Cancer Registries, which was 20.4 ([2], their reference 28).

Further, Piscitelli et al.′s assertion that “the incidence of all pediatric cancer in Italy (is) about 11,800 new cases per year” [1] finds no support from their own data.

Cancer in children causes a major emotional impact, particularly in highly polluted areas such as some Italian provinces. Influencing risk perception through the circulation of unreliable epidemiological data seems to us out of place.
